# Direct phase measurement of waveguides with a next generation optical vector spectrum analyzer

**DOI:** 10.1038/s41377-024-01574-3

**Published:** 2024-10-19

**Authors:** Andrew Grieco

**Affiliations:** Qualcomm Institute, University of California San Diego, 9500 Gilman Dr, La Jolla, CA 92093 USA

**Keywords:** Optical spectroscopy, Frequency combs, Imaging and sensing

## Abstract

A novel dual-mode optical vector spectrum analyzer is demonstrated that is suitable for the characterization of both passive devices as well as active laser sources. It can measure loss, phase response, and dispersion properties over a broad bandwidth, with high resolution and dynamic range.

Spectroscopy is one of the most critical characterization technologies for scientific research and industrial engineering^1^. Recently, the field has undergone significant development due to growth in the application space that has created strong demand for devices with improved performance, reduced power consumption, and small footprint^2,3^. As the device platforms become miniaturized, the manifestation of dispersive effects becomes highly significant^4^. This consideration is particularly important in the context of integrated optics^5^. In the presence of these effects careful frequency calibration is required, which can be performed using an optical vector network analyzer.

Optical vector network analyzers characterize the transmission, loss, phase response, and dispersion of a passive device by measuring the linear transfer function^6^. Conventional designs can be broadly grouped into three operating principles: interferometric^7^, optical channel estimation^8,9^, and sideband modulation^10,11^. The sideband modulation category can further be broken down into single-sideband and double-sideband devices. Interferometric devices utilize a tunable laser in conjunction with a combination of interferometers, one of which contains the device under test. The transfer function is extracted by performing signal processing on the interferogram. Optical channel estimation devices operate similarly to coherent optical orthogonal frequency-division multiplexing systems, where comparison between transmitted and received symbols is used to infer the transfer function. Sideband modulation devices operate by using an optical passband filter and a modulated probe laser incident on the device being tested to generate an asymmetric output signal that encodes the transfer function of the device, which can then be reconstructed. In terms of performance, these techniques can achieve a high resolution, however their bandwidth is limited. This renders them unsuitable for characterization of broadband devices such as optical frequency combs, parametric oscillators/amplifiers, and supercontinuum sources.

In a recently published paper in *Light: Science & Applications*, a research team led by Professor Junqiu Liu from the Shenzhen International Quantum Academy has developed a novel dual-mode optical vector spectrum analyzer that is capable of characterizing the transfer function of passive devices, as well as perform analysis on optical sources by coherently mapping an optical spectrum into the radio frequency domain^12^. It operates over a broad bandwidth, with high resolution and dynamic range. In this design a chirped laser is either transmitted through a device being tested, or interfered with an optical source being tested. In the case of the device, the transfer function can be extracted by interfering the transmitted signal with that from a reference path. In the case of the optical source, the interference produces a progressive radio frequency beatnote signal from which the spectral information can be recovered. In both cases the output signal is a time-domain interferogram from which the frequency-domain response can be recovered using digital signal processing. The success of this approach requires precision calibration of the chirped laser frequency, which is accomplished by the incorporation of a fiber resonant cavity to acts as a frequency ruler. Remarkably the system does not incorporate any sort of high-speed modulators/detectors, and it does not require any active feedback mechanism (see Fig. 1 in the original manuscript^12^ for a schematic of the device in the configuration for characterizing passive devices, which is reproduced here). The design itself is highly extensible, and can be adapted to other spectral ranges by the addition of appropriate chirped lasers.

**Fig. 1:**
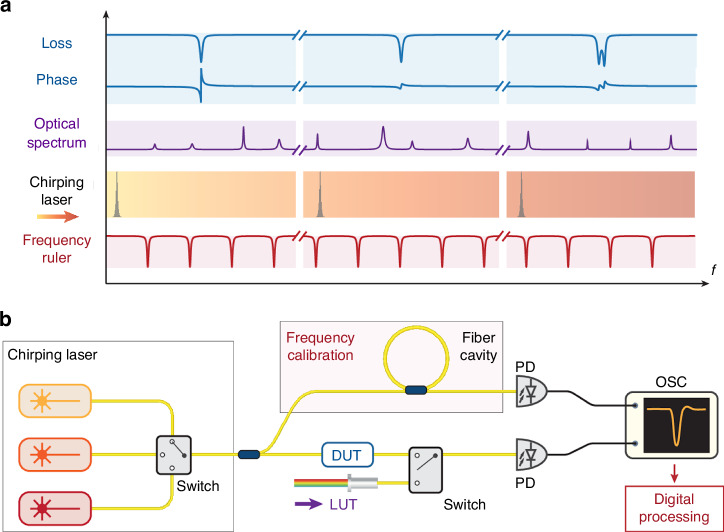
Schematic of the optical vector network analyzer. **a** The principle of our VSA is based on a chirping CW laser that is sent to and transmits through a device under test (DUT), or interferes with any light under test (LUT). The DUT can be any passive device, and the LUT can be any broadband laser source. The transmission spectrum of the chirping laser through the DUT, and the beatnote signal generated from the interference between the chirping laser and the LUT, are both time-domain traces. Together with a “frequency ruler” for calibration, the chirping laser coherently maps the time-domain trace into the frequency domain. This trace carries the information of the DUT’s loss, phase and dispersion over the chirp bandwidth (blue). For LUT, the chirping laser beats progressively with different frequency components of the optical spectrum, thus, analyzing the beat signal in the RF domain allows extraction of the spectral information (purple). Critical to this frequency-time mapping is precise and accurate calibration of the instantaneous laser frequency during chirping. This requires referring the chirping laser to the frequency ruler (red). **b** Experimental setup. The chirping laser unit can be a single laser, or multiple lasers that are bandwidth-cascaded. The latter allows extension of the full spectral bandwidth by seamless stitching of individual laser traces into one trace. The chirping laser power is then split into two branches. The upper branch is directed to the frequency-calibration unit (i.e. the “frequency ruler”), which in our case, is a phase-stable fiber cavity of 55.58 MHz FSR. The lower branch is sent to the DUT or the LUT. For the two branches, the photodetector signals are recorded with an oscilloscope and digitally processed offline. PD photodetector, OSC oscilloscope. Note that the figure and caption are reproduced from the original manuscript^12^

Finally, in the paper, it is experimentally demonstrated that with minor modifications the base vector spectrum analyzer system can be employed to additional applications, including light detection and ranging (LiDAR) and mapping frequency comb spectra. It is also speculated that the system can be applied to “… time-stretched systems^13^, optimized optical coherent tomography (OCT)^14^, linearization of FMCW LiDAR^15^, and resolving fine structures in Doppler-free spectroscopy^16^.” This serves to underscore the utility of the design approach, and suggests exciting directions for future research.
